# Significant Increase in Cytotoxic T Lymphocytes and Natural Killer Cells by Triphala: A Clinical Phase I Study

**DOI:** 10.1155/2012/239856

**Published:** 2012-12-02

**Authors:** Pratya Phetkate, Tanawan Kummalue, Yaowalak U-pratya, Somboon Kietinun

**Affiliations:** ^1^Faculty of Medicine, Thammasat University, Pathumthani 12121, Thailand; ^2^Department of Clinical Pathology, Faculty of Medicine Siriraj Hospital, Mahidol University, Bangkok 10700, Thailand; ^3^Division of Hematology, Department of Medicine, Faculty of Medicine Siriraj Hospital, Mahidol University, Bangkok 10700, Thailand

## Abstract

*Background*. Searching for drugs or herbal formulations to improve the immunity of HIV/AIDS positive people is an important issue for researchers in this field. Triphala, a Thai herbal formulation, is reported to have immunomodulatory effects in mice. However, it has not yet been investigated for immunostimulatory and side effects in healthy human volunteers. *Objective*. To evaluate the immunostimulatory and side effects of Triphala in a clinical phase I study. *Materials and Methods*. All volunteers took Triphala, 3 capsules per day for 2 weeks. Complete physical examination, routine laboratory analysis, and immunological studies were performed before ingestion and after initial meeting for 4 consecutive weeks. *Results*. We found that Triphala demonstrated significant immunostimulatory effects on cytotoxic T cells (CD3^−^CD8^+^) and natural killer cells (CD16^+^CD56^+^). Both of them increased significantly when compared with those of the control samples. However, no significant change in cytokine secretion was detected. All volunteers were healthy and showed no adverse effects throughout the duration of the study. *Conclusion*. Triphala has significant immunostimulatory effects on cellular immune response, especially cytotoxic T cells and natural killer cells. Increases in the absolute number of these cells may provide a novel adjuvant therapy for HIV/AIDS positive people in terms of immunological improvement.

## 1. Introduction

The number of people with HIV/AIDS positive blood, a disease caused by Human immunodeficiency (HIV) virus, has increased to approximately 5.6 million people around the world as reported in 2010, though HIV/AIDS incidence has declined in some countries including Thailand [1]. Life expectancy for HIV positive adults depends on sociodemographic factors and the level of immunosuppression [2]. As is well known, the important pathophysiology of this disease is caused by immune system dysfunction especially depletion of CD4^+^ T cells with consequent opportunistic infections and diseases, such as cryptococcal meningitis, multidrug-resistant tuberculosis, and *Pneumocystis carinii* pneumonia. It has been shown that cell-mediated immune responses through cytotoxic T lymphocytes (CTLs) and natural killer (NK) cells play an important role in host defense against viral infection and also against opportunistic infections in HIV. Both CTL and NK cells have the ability to suppress HIV replication in autologous CD4^+^ T cells [3]. Moreover, NK cells with *KIR3DS1/HLA-B Bw4-80I* genotype confer protection against the development of AIDS opportunistic infections [4]. Therefore, the immunomodulatory drugs or herbal formulations to improve cellular-mediated immunity for treating HIV/AIDS have been intensively researched to achieve the high quality care for this HIV/AIDS group.

Triphala (meaning three fruits) is a Thai traditional herbal formulation in Thai Herbal Pharmacopoeia originating in India. It is composed of; *Terminalia chebula* Retz., in the family Combretaceae; *Terminalia belerica* Linn., in the family Combretaceae; *Phyllanthus emblica* Linn., in the family Euphorbiaceae. From phytochemical analysis, Triphala was demonstrated to have several chemical compounds in the megaext mixture, namely, alkaloids, carbohydrates, glycosides, terpenoids, tannins, phenolic compounds, flavonoids, and proteins, and so forth [5]. Triphala has long been prescribed by traditional doctors for treating various diseases, such as cardiovascular diseases including high blood pressure. It is also used to alleviate several symptoms, such as digestive problems, and constipation [6]. In Thailand, it is traditionally used for adjusting the body elements to climate change for strength and healthiness. In short, from the ancient knowledge of Thai traditional medicine, the human body is composed of 4 basic elements, namely, the earth, water, air, and fire elements. Alterations in these 4 elements can induce discomfort and sickness.

Based on several scientific studies, Triphala, in the equal proportion formulation (1 : 1 : 1), has demonstrated many effects in mice, that is, chemoprotection, anti-inflammation, and immunomodulation [7, 8]. The anticancer activity of Triphala has also been investigated; Triphala has been found to inhibit growth of several malignancies including both in vitro and in vivo, such as breast cancer, prostate cancer, and pancreatic tumor [9–11]. A recent report demonstrates that Triphala can mediate its antitumor effects via inhibition of vascular endothelial growth factor and can prevent angiogenesis [12]. 

Triphala has been reported to improve immunological status, especially immunostimulatory effects in mice. The effects of Triphala on phagocytic cell function, such as the neutrophil function, have also been studied. Researchers have found that Triphala stimulated neutrophil function without increasing the number in a study of mice under conditions of induced noise stress [13]. Interestingly after sensitization with sheep red blood cells (SRBCs) antigen in mice, activation of T cells and cytokine secretion in delayed type hypersensitivity response was demonstrated in Triphala-treated mice when compared with mice in the control group [5]. However, the clinical phase I study of Triphala has not yet been reported. Therefore, this prompted us to investigate the clinical phase I of Triphala in healthy volunteers to evaluate the immunomodulatory effects and side effects of this well-known herbal formulation for determining its immunotherapeutic potential in the treatment of HIV/AIDS.

## 2. Materials and Methods

### 2.1. Plant Preparation and Standardization

Triphala with equal proportions of 3 plant materials in a mixture of 1 : 1 : 1 (w/w) was prepared. It was macerated with 95% ethanol by the Herb and Food Research Center at the Faculty of Medicine at Thammasat University. The percentage yield obtained was 34 grams. At the same center, the standardization of Triphala was performed by high performance liquid chromatography (HPLC) using gallic acid as the marker compound.

### 2.2. Triphala Dosage Calculation

 According to the “Thai List of Herbal Medicinal Products,” Triphala, in powder preparation, is usually prescribed at a dosage of 1-2 grams by adding it to 200 mL of drinking water every 4 hours [14]. The NOAEL (no-observed-adverse-effect level) at 2,400 mg/kg/d was determined by the Animal Research Division of the Faculty of Medicine at Thammasat University. Using NOAEL and probabilistic multiplication, a reference dose (RfD) of Triphala was calculated and used in this study [15]. The formulation is shown below, while UF_H_ and UF_S_ mean interspecies variability and intraspecies variability, respectively,

(1) $$\begin{array}{c} \text{RfD}=\text{NOAEL}\times \text{Body weight}÷\left(\text{U}{\text{F}}_{\text{H}}\times \text{U}{\text{F}}_{\text{S}}\right). \end{array}$$

As calculated, an extract not exceeding 1,200 mg was determined to be an effective and safe daily dosage for human ingestion. Regarding the percentage yield, each capsule, produced by CDIP (Thailand) Co., Ltd., contained 350 mg of the extract mentioned earlier. 

### 2.3. Healthy Volunteers Clinical Studies

 The 20 volunteers were divided into groups of males and females with 10 persons in each group. Inclusion criteria for healthy volunteers were healthy males and nonpregnant females between 20 and 45 years of age with no present medical illness and no present ingestion of medication or any herbal formulation. All volunteers took 1 capsule (350 mg) with meals three times daily (1,050 mg/day) for 2 weeks (D0–D14). Physical examination, routine laboratory analysis, and immunological studies were done before and after ingestion for 4 consecutive weeks. The schematic diagram of this study was demonstrated in Figure 2.

This study was conducted in accordance with the Declaration of Helsinki and approved by the Human Ethics Committee of Thammasat University. All volunteers provided written informed consent.

### 2.4. Routine Laboratory Analysis

 Venous blood was drawn and examined for complete blood count; renal function test, namely, blood urea nitrogen, and creatinine; fasting blood sugar; liver function tests such as SGOT, SGPT, alkaline phosphatase, bilirubin, albumin, and globulin; lipid profiles including cholesterol, triglyceride, and HDL; and urine examination. These routine laboratory analyses were performed by the Bangkok Pathology-Laboratory with automated equipment.

### 2.5. Immunological Studies for Expression of Surface Markers

 The fresh EDTA 3 mL venous blood was examined for immunological profiles including CD3, CD4, CD8, CD45, CD19, CD16, CD56, CD25, and CD69 (BD, Biosciences, USA). In brief, 50 *μ*L of EDTA blood was incubated with 20 *μ*L of MultiTest CD3 FITC/CD8 PE/CD45 PerCP/CD4 APC, MultiTest CD3 FITC/CD 16 + 56 PE/CD45 PerCP/CD19 APC, or TriTest Control *γ*_1_ FITC/*γ*_1_ PE/CD45 PerCP in a separated tube and 10 *μ*L of each antibody including CD4 FITC, CD69 PE, CD45 PerCP, and CD25 APC at 4°C in darkness for 30 minutes, followed with 2 mL of FACSlysing reagent (BD, Biosciences, USA) at room temperature for 10 minutes. The white blood cells were washed twice with cold PBS (Sigma, Thailand) and resuspended with 0.5 mL of 1% paraformaldehyde in PBS. The acquisition and analysis were performed using FACSCalibur and CellQuest software.

### 2.6. Cytokine Secretion Measurement

 Interleukin-6 (IL-6), interferon-*γ* (IFN-*γ*), and tumor necrosis factor-*α* (TNF-*α*) were detected from volunteers' serum using Human ELISA kit (BD, Biosciences, USA) following the manufacturer's instructions. In brief, after centrifugation of the clotted blood for 10 minutes at 1000 xg, the serum was carefully collected and kept at −20°C before usage. The standard curve was plotted for determination of results. The well-prepared standard reagent and samples were then added into each well in 96 well plates, followed by the working detector, TMB one-step substrate reagent, and finally with a stop solution. Results were determined using the ELISA reader (Biochrom, UK) at a wavelength of 450 nm within 30 minutes.

### 2.7. Statistical Analysis

The results were expressed as mean ± SD. The statistical analysis was conducted using pair *t*-test and SPSS software. A *P*  value < 0.05 was considered statistically significant.

## 3. Results

### 3.1. Physical Examination and Routine Laboratory Analysis

 All volunteers were healthy, and no adverse effects were detected during the 4-week period. The routine laboratory data of these volunteers was in normal (reference) ranges throughout the duration of this study. No significant change in any routine laboratory parameter was found during this clinical phase I study.

### 3.2. Activation of Expression of Surface Markers

**Figure 1:**
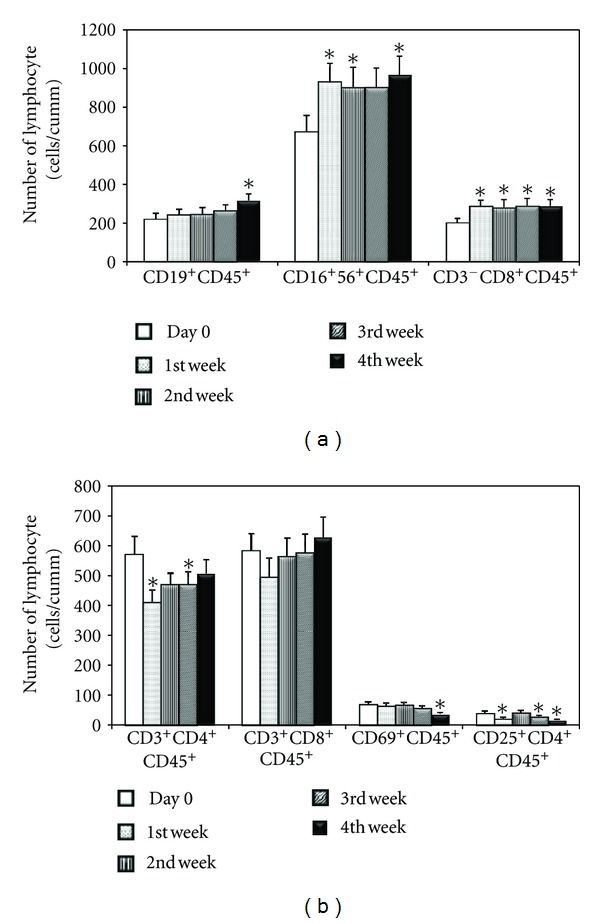
The absolute numbers of lymphocyte subpopulations before and after Triphala ingestion were shown. (a) The marked increase in CD16^+^56^+^CD45^+^ cells together with CD3^−^CD8^+^CD45^+^ cells was found (*P < 0.05) after the 1st throughout the 4th week of this clinical phase. The CD19^+^CD45^+^ B lymphocytes showed significantly increase after the 4th week (*P < 0.05). (b) The significant decrease of CD3^+^CD4^+^CD45^+^, CD69^+^CD45^+^, and CD25^+^CD4^+^CD45^+^ T cells were found after Tripala ingestion (*P < 0.05). There was no significant change in the absolute numbers of CD3^+^CD45^+^ cells. The data were presented as mean ± SEM, *n* = 20.

**Figure 2:**
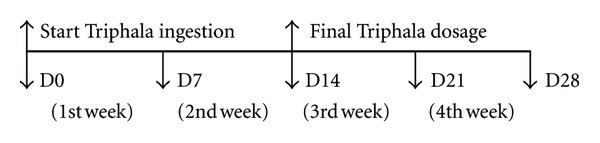
D0: the first day of Triphala ingestion. Complete physical examination including routine laboratory analysis and immunological studies were first performed on this day. D28: the final follow-up day included physical examination, routine laboratory analysis, and immunological studies.

Immunological exploration including NK, T, and B cell populations were investigated in this clinical phase. Strikingly, the absolute number of cytotoxic T cells expressed CD3^−^CD8^+^CD45^+^ surface markers was significantly increased when compared with the cells expressed in the control samples with the *P*  value < 0.05. This significant evidence could be detected from the first week (about 7 days) after Triphala ingestion. It was also noted that CD16^+^56^+^CD45^+^ expressed cells, the surface markers of natural killer cells, were significantly elevated after the first week of this clinical study (as shown in Figure 1 and Table 1). Importantly, this absolute number of NK cells showed sustained elevation until the last week of the 4-week period. Moreover, the absolute number of CD19^+^CD45^+^ B lymphocytes showed significant elevation at the fourth week (as shown in Figure 1).

 In contrast, CD25^+^CD4^+^CD45^+^ T cells, known as regulatory T cells and CD69^+^CD45^+^ T cells, steadily decreased during the third and fourth weeks of Triphala ingestion, respectively. In a similar pattern, the absolute number of CD3^+^CD4^+^CD45^+^ T cells showed a transiently significant decrease during the first and third weeks in this study (as shown in Figure 1). 

### 3.3. Cytokine Assay

Using ELISA technology, no significant elevation of IL-6, IFN-*γ*, and TNF-*α* was noticed in the volunteers' serum throughout this clinical phase.

## 4. Discussion

This clinical phase I study aimed to evaluate the immunomodulatory and side effects of Triphala. As mentioned earlier, the life expectancy of HIV/AIDS positive persons depends on their immunological status. The greater their immunological improvement, the longer they live as normal lives. Therefore, searching for drugs or herbal formulations to improve the immune system is one of the primary objectives of researchers.

 Based on this clinical phase I study, regarding its safety, Triphala with equal proportions (1 : 1 : 1) has been shown to be safe for use in healthy volunteers with a dosage of 1,050 mg per day; a follow-up test was performed two weeks after finishing the final dosage. No side effects or adverse effects were detected by physical examination and routine laboratory analysis. The blood samples from all volunteers showed no change in liver function test, renal function test, fasting blood sugar, lipid profiles, or complete blood count compared to the control group. No specific organ damage was found, including damage to liver, kidney, pancreas, or bone marrow.

 The most important findings of this study were the significant increases in cytotoxic T lymphocytes, natural killer cells, and B lymphocytes. As known, HIV/AIDS positive people have immunosuppression because of a decreased CD4^+^ T cell population including both naïve and memory T cells due to viral infection [16]. Cytotoxic T lymphocyte (CTL) with the CD8^+^ surface marker expression is the critical portion in cell-mediated immunity to confront virally infected cells. By using the MHC class I processes and releasing the toxic perforin and granzymes from its cytoplasm, the CTL can then effectively lyse and kill the virally infected cells [17]. Therefore, increasing the absolute number of CTLs by consuming Triphala might yield improvements and increments in the cytolytic process; consequently, this might help reducing the viral load in an HIV/AIDS positive person. 

Natural killer (NK) cells play a pivotal role in innate immunity in conjunction with adaptive immunity. Their functions depend on distinguishing the MHC class I positive cells from others, such as those infected with virus. By using specific receptors on the cell surface, NK cells can kill virally infected cells. In HIV/AIDS, the cytolytic functional defects of NK cells due to downregulation of activating NK receptors, NKG2A, have been reported [18]. In this study, Triphala significantly increased the absolute number of NK cells from the first week. These cells were highly sustained until the final week of this clinical phase. Taken together, it is possible that Triphala could rapidly improve innate immunity, especially cellular components, which would be beneficial for treating HIV/AIDS. 

The increase in the number of cytotoxic T lymphocytes and natural killer cells by Triphala may derive from the immunostimulatory action of all three fruits, namely, *Phyllanthus emblica*, *Terminalia chebula, and Terminalia belerica. *Previous reports showed that *Phyllanthus emblica* can enhance immunity both in vitro and in vivo, especially in NK cell-induced cytotoxic activity [19, 20]. Its fruit is rich in various chemical compounds including gallic acid, vitamin C, and quercetin. Recent reports demonstrated that quercetin can significantly increase immunity in rats, whereas vitamin C plays an immunomodulatory role by increasing cell-mediated immune response. Vitamin C can improve several components of human immune parameters such as lymphocyte proliferation and function plus NK cell activities [21, 22]. *Terminalia chebula*, with chebulinic acid as a principal constituent, can increase T and B lymphocytes and also activate NK cells in animal studies [23, 24]. In addition, *Terminalia belerica *has been reported to stimulate T lymphocytes in mouse studies [25]. 

No significant change in cytokine secretion was detected in this clinical study. The reason for this phenomenon might be explained by the healthy status of the volunteers themselves. In this regard, NK cells and macrophages were not activated by the real pathogens or virus infected cells in the volunteers. Therefore, an immune response was not initiated or activated. In this aspect, no cytokine was released from the inactivated NK cells and macrophages.

CD19^+^CD45^+^ B lymphocyte elevation was detected after finishing the 14-day period of Triphala ingestion. Further, there may be a tendency for an increase in these cells after the fourth week of the study. B lymphocytes play an essential role in humoral immunity by producing antibodies to coat and destroy foreign substances including infected cells. Based on this study, a significant increase in B lymphocytes by consuming Triphala might increase humoral immunity and improve cell-mediated immunity in NK cell function; this is especially relevant concerning the antibody-dependent cellular cytotoxicity (ADCC) pathway. An ADCC response refers to the way that antibodies target viral proteins which are present on the surface of infected cells. Subsequently, these infected cells will be recognized and killed by NK cells. However, the success of humoral immunity and the NK-ADCC response to suppress HIV remains uncertain [26–28]. 

On the contrary, the significant and stable decrease in the absolute number of CD25^+^CD4^+^CD45^+^ T cell group, known as regulatory T (Treg) cells, was found at the third week. This T cell subset plays an important role in the suppression of the immune response and has been found to infiltrate tumors of many human malignancies. Treg-infiltrated tumors are often associated with poor clinical outcomes [29]. In this special infection of HIV/AIDS, Treg can suppress T cell response and thus inhibit HIV replication in T cells [30]. However, the beneficial role of Treg in this situation has only recently been reported. Increases in this T cell subset could, therefore, aggravate its inhibitory function on other immune cells; consequently, it could suppress the immune response as well. In this investigation, CD3^+^CD4^+^CD45^+^ T cells were demonstrated to decrease transiently. It should be pointed out that this decrement might result from increases in other T cell subsets, including NK cells, and CTLs. This may cause the relative decrease in the rest of the T cell population while the total number of white blood cells remains constant. 

A significant decrease in CD69^+^CD45^+^ T cells as activation markers on lymphocytic surfaces was also demonstrated during the fourth week of this study. This specific CD69^+^ expression is associated with acute activation of T cells. Recently, this surface marker was found to be critical for the persistence of CD4^+^ T cells memory in the bone marrow environment [31]. Based on this study, Triphala ingestion for 2 weeks might not be sufficient to activate the number and function of T lymphocyte subset, especially CD4^+^ T cells that play an important role in adaptive immunity.

In conclusion, this clinical phase I study with Triphala ingestion for 2 weeks demonstrated that Triphala can serve as a potential immunostimulatory herbal formulation for HIV/AIDS positive persons. The safety of the herb plus the significant increase in cytotoxic T cells, NK cells, and B lymphocytes might be beneficial to improve the immune system; this applies to both innate and adaptive immunities for high quality and long life expectancy. Indeed, the duration of Triphala ingestion, if used as an immunomodulatory therapy in HIV/AIDS, should take longer than the time estimated by the pilot survey in this study. Long-term usage and followup including the functional assay of lymphocytes might require further investigation.

## Figures and Tables

**Table 1:** The percentage of lymphocyte subpopulations before and after Triphala ingestion (*n* = 20).

Cell types	The percentage of lymphocyte (mean ± SD)				
	D0	D7	D14	D21	D28
CD3^+^CD45^+^	60.56 ± 14.57	48.78 ± 12.45^a^	55.61 ± 13.22	53.82 ± 11.43^a^	53.06 ± 10.98^a^
CD3^+^CD4^+^CD45^+^	25.79 ± 9.42	18.65 ± 6.02^a^	21.49 ± 6.72^a^	20.46 ± 6.29^a^	19.97 ± 5.60^a^
CD3^+^CD8^+^CD45^+^	26.62 ± 10.21	22.77 ± 10.33^a^	25.56 ± 11.08	25.28 ± 10.91	24.73 ± 10.02
CD3^−^CD8^+^CD45^+^	9.48 ± 5.20	12.76 ± 5.22	11.94 ± 6.14^b^	12.10 ± 5.50	10.67 ± 4.20
CD19^+^CD45^+^	9.94 ± 4.46	11.41 ± 5.73	11.27 ± 5.58	11.56 ± 5.0^b^	12.55 ± 5.56^b^
CD16^+^56^+^CD45^+^	30.64 ± 15.34	41.16 ± 12.81^b^	38.72 ± 12.97^b^	38.12 ± 10.62^b^	36.88 ± 9.84
CD25^+^CD4^+^CD45^+^	1.88 ± 1.30	0.96 ± 0.54^a^	2.12 ± 2.07	1.31 ± 0.88^a^	0.69 ± 0.32^a^
CD69^+^CD45^+^	3.27 ± 1.50	3.06 ± 1.62	3.19 ± 1.74	2.60 ± 1.19^a^	1.52 ± 0.69^a^

^a^Significant decreased, *P* < 0.05; ^b^Significant increased, *P* < 0.05.
